# Body mass index and COVID-19 mortality: prospective study of 120 000 Mexican adults

**DOI:** 10.1093/ije/dyac134

**Published:** 2022-10-13

**Authors:** Jesus Alegre-Díaz, Louisa G Friedrichs, Raul Ramirez-Reyes, Rachel Wade, Fiona Bragg, William G Herrington, Robert Clarke, Richard Peto, Rory Collins, Pablo Kuri-Morales, Jonathan R Emberson, Roberto Tapia-Conyer

**Affiliations:** 1 School of Medicine, National Autonomous University of Mexico, Mexico City, Mexico; 2 MRC Population Health Research Unit, Nuffield Department of Population Health, University of Oxford, Oxford, UK; 3 Clinical Trial Service Unit & Epidemiological Studies Unit, Nuffield Department of Population Health, University of Oxford, Oxford, UK

**Keywords:** BMI, COVID-19, Mexico, prospective study

Worldwide, there have already been about 20 million excess deaths during the coronavirus disease 2019 (COVID-19) pandemic (based on a recent estimate of 18 million during 2020-21), but only 6 million deaths have been individually attributed to COVID-19, suggesting 3-fold under-attribution.^1,2^ In Mexico the excess number of deaths during 2020–21 has been estimated as 0.8 million, but only 0.3 million deaths have been individually attributed to COVID-19, again suggesting substantial under-attribution. Within Mexico City, however, the attribution of causes of death may well have been much more reliable.

Obesity and obesity-related diseases are major risk factors for COVID-19 mortality. In the UK, where the Office for National Statistics registration of COVID-19 deaths has been reasonably accurate since May 2020, a study of 7 million adults reported that each 5-kg/m^2^ higher body mass index (BMI) above 23 kg/m^2^ was associated with a 28% higher risk of death from COVID-19, but the association appeared to be ‘J-shaped‘.^3^ In Mexico, where the prevalences of overweight and obesity are among the highest in the world,^4^ a study of 50 000 adults attending hospital, who tested positive for SARS-Cov-2 using polymerase chain reaction, found that self-reported obesity (yes versus no) was associated with a 31% higher case-fatality rate; but the study did not have data on BMI and so could not assess the shape of the association of BMI with case-fatality.^5^ Using the Mexico City Prospective Study (MCPS) of adult mortality in the general population, we report the association between BMI and COVID-19 mortality throughout the full range from normal weight to morbidly obese, comparing this with similar analyses of mortality from all other causes combined.

The MCPS recruited 150 000 adults in 1998-2004, measured BMI and other factors and followed participants through the death registry for cause-specific mortality.^6^ Death registration in Mexico City is reliable, with almost all adult deaths certified medically.^7^ Deaths due to COVID-19 were defined as those with an International Classification of Diseases 10th revision (ICD-10) underlying cause U07.1 or U07.2. Among survivors aged <90 years on 1 January 2020, we used Cox regression to relate baseline BMI (which correlates strongly with BMI many years later^8^) to 2020 COVID-19 mortality. Associations with BMI were estimated across six categories (from normal to morbidly obese, excluding participants with BMI outside the range 18.5–60 kg/m^2^), adjusted for age, sex, residential district and highest educational level. (The few participants with missing data for BMI or any confounder were excluded.) Effects per 5-kg/m^2^ higher BMI were also reported, overall, by sex and by age (<70 vs ≥70 years on 1 January 2020). Analyses used SAS version 9.4 (SAS Institute) and R version 3.1.1 [www.r-project.org/]. Data were accurate as of May 2022.

Among the 122 022 participants (37 720 men and 84 302 women) alive on 1 January 2020 [mean age 67 (SD 10) years, 65% under age 70], BMI at baseline was 28.1 (SD 4.0) kg/m^2^ in men and 29.6 (5.1) kg/m^2^ in women; 28% of men and 42% of women had a BMI >30 kg/m^2^ (Table 1). University or college education was more common in younger than older individuals and more common in men than women. During 2020, 678 died from COVID-19 and 1572 from other underlying causes. BMI was positively (and approximately log-linearly) associated with COVID-19 mortality, with no evidence of any threshold level below which lower BMI was not associated with lower risk (Figure 1). In the lowest BMI category, COVID-19 mortality among participants with BMI 22.5–25 kg/m^2^ (mean 24 kg/m^2^) was nearly twice that among those with BMI 18.5–22.5 kg/m^2^ (mean 21 kg/m^2^): mortality rate ratio (RR) 1.89, but with 95% confidence interval (CI) 1.01–3.53. Across the whole range, each 5-kg/m^2^ higher BMI was associated with 42% higher COVID-19 mortality (RR 1.42 per 5-kg/m^2^, 1.32–1.52). Comparing the top and bottom categories (BMI ≥40 versus <25 kg/m^2^, with means 43 kg/m^2^ and 23 kg/m^2^, respectively), there was about a 4-fold difference in COVID-19 mortality (RR 4.47, 2.98–6.70). The association was stronger in younger than older adults (RR per 5-kg/m^2^ higher BMI 1.61, 1.47–1.76, if age <70 years on 1 January 2020 versus 1.18, 1.05–1.32, if older) but, at any given age, was comparable in men and women. BMI was also strongly predictive of mortality from the aggregate of all other causes, with each 5-kg/m^2^ higher BMI associated with 32% higher mortality (RR 1.32, 1.26–1.39). In a sensitivity analysis restricted to the 348 deaths with an ICD-10 code of U07.1 (i.e. COVID-19 where the virus was confirmed by laboratory testing) the RR per 5 kg/m^2^ higher BMI was 1.36 (1.22–1.50).

Overweight (BMI 25–30 kg/m^2^) and obesity (BMI ≥30 kg/m^2^) affect more than one-third of the global adult population.^9^ Our findings highlight the importance of overweight in addition to obesity as a major modifiable risk factor for death from COVID-19. However, even during 2020 other causes of death accounted for more than twice as many deaths as COVID-19, so the absolute association of BMI with mortality was greater for the aggregate of those other causes than for COVID-19. Considering not only the pandemic but also other years, these other causes are the main way obesity reduces life expectancy. In addition, as overweight is much more common than morbid obesity, its population effects are more important.

## Figures and Tables

**Figure 1:**
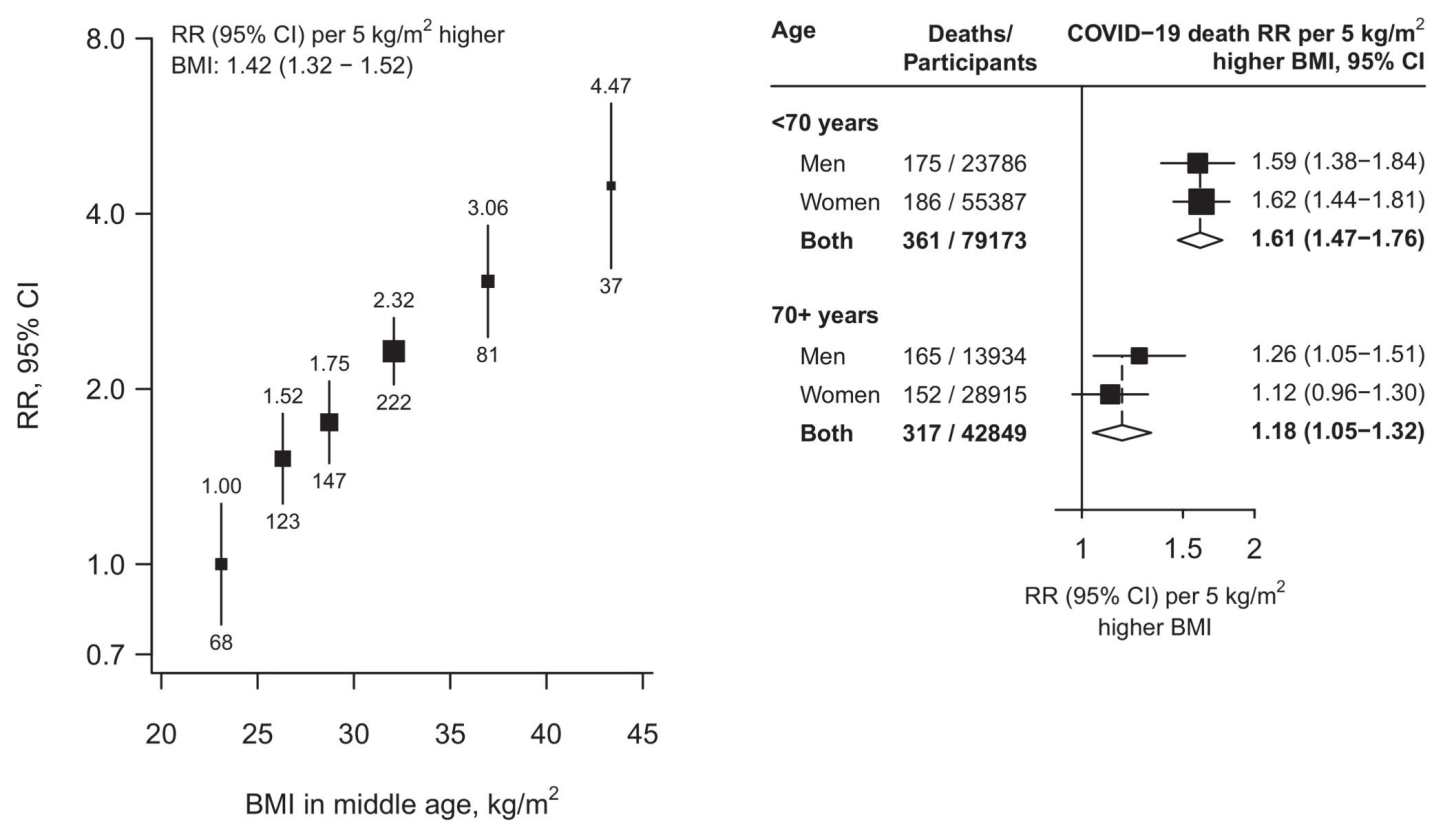
Body mass index versus risk of death from COVID-19. In the left panel, estimates are adjusted for age, sex, education and district and in the right panel, the age- and sex-stratified results are adjusted for age, education and district. The areas of the squares are proportional to the amount of statistical information and the lines through them are the 95% confidence intervals. The numbers above each vertical line in the left panel show the death rate ratio for that group and the numbers below each vertical line give the number of COVID—19 deaths in that group. In the right panel, the diamonds are the information-weighted averages of the two results above them. BMI, body mass index; CI, confidence interval; RR, mortality rate ratio

**Table 1:** Characteristics of 122 022 individuals from the Mexico City Prospective Study aged <90 years on 1 January 2020

	Men		Women	
	<70 years (*n* = 23 786)	≥70 years (*n* = 13 934)	<70 years (*n* = 55 387)	≥70 years (*n* = 28 915)
Age, years	61 (5)	78 (6)	61 (5)	78 (6)
Resident of Coyoacán	10 040 (42%)	6824 (49%)	20 021 (36%)	12 782 (44%)
University or college educated	8212 (35%)	2317 (17%)	9535 (17%)	1676 (6%)
Body mass index (kg/m^2^) at original study recruitment (1998–2004)			
Mean (SD)	28.1 (4.1)	28.1 (3.9)	29.4 (5.1)	30.0 (5.0)
≥18.5 to <25	5191 (22%)	3000 (22%)	10 345 (19%)	4278 (15%)
≥25 to <30	11783 (50%)	7026 (50%)	22 937 (41%)	11 344 (39%)
≥30	6812 (29%)	3908 (28%)	22 105 (40%)	13 293 (46%)

Numbers shown are mean (standard deviation) or *n* (%).

## Data Availability

We welcome requests from researchers who wish to access data from the Mexico City Prospective Study. If you are interested in obtaining data from the study for research purposes, or in collaborating with us on a specific research proposal, please visit our study website [https://www.ctsu.ox.ac.uk/research/prospective-blood-based-study-of-150-000-individuals-in-mexico] where you can download our Data and Sample Access Policy in either English or Spanish.
